# Crystal structures of cables formed by the acetylated and unacetylated forms of the *Schizosaccharomyces pombe* tropomyosin ortholog Tpm^Cdc8^

**DOI:** 10.1016/j.jbc.2024.107925

**Published:** 2024-10-25

**Authors:** Patrick Y.A. Reinke, Robin S. Heiringhoff, Theresia Reindl, Karen Baker, Manuel H. Taft, Alke Meents, Daniel P. Mulvihill, Owen R. Davies, Roman Fedorov, Michael Zahn, Dietmar J. Manstein

**Affiliations:** 1 Institute for Biophysical Chemistry, Fritz–Hartmann–Centre for Medical Research, Hannover Medical School, Hannover, Germany; 2 Division for Structural Biochemistry, Hannover Medical School, Hannover, Germany; 3 FS–BMX, Deutsches Elektronen–Synchrotron DESY, Hamburg, Germany; 4 School of Biosciences, University of Kent, Canterbury, Kent, UK; 5 Wellcome Centre for Cell Biology, Institute of Cell Biology, University of Edinburgh, Michael Swann Building, Max Born Crescent, Edinburgh, UK

**Keywords:** actin, Cdc8p, tropomyosin overlap junction, acetylation, cell motility, cytoskeleton, *Schizosaccharomyces pombe*

## Abstract

Cables formed by head-to-tail polymerization of tropomyosin, localized along the length of sarcomeric and cytoskeletal actin filaments, play a key role in regulating a wide range of motile and contractile processes. The stability of tropomyosin cables, their interaction with actin filaments and the functional properties of the resulting co-filaments are thought to be affected by N-terminal acetylation of tropomyosin. Here, we present high–resolution structures of cables formed by acetylated and unacetylated *Schizosaccharomyces pombe* tropomyosin ortholog Tpm^Cdc8^. The crystal structures represent different types of cables, each consisting of Tpm^Cdc8^ homodimers in a different conformation. The structures show how the interactions of the residues in the overlap junction contribute to cable formation and how local structural perturbations affect the conformational dynamics of the protein and its ability to transmit allosteric signals. In particular, N-terminal acetylation increases the helicity of the adjacent region, which leads to a local reduction in conformational dynamics and consequently to less fraying of the N-terminal region. This creates a more consistent complementary surface facilitating the formation of specific interactions across the overlap junction.

The first experimental evidence for the existence of coiled–coiled protein structures and a comprehensive description of the underlying design principles dates back to Crick's pioneering work in the 1950s. The work included the first description of tropomyosin (Tpm) as a two-stranded parallel coiled–coil that binds actin filaments in a head–to–tail arrangement by forming quasi–continuous cables winding around the length of the filament (1, 2, 3). Canonical parallel two-stranded α–helical coiled–coils, as shown in Fig. 1*A*, are formed from polypeptide chains possessing a typical heptad repeat pattern with hydrophobic residues at positions *a* and *d*, thereby forming a hydrophobic core with knob into hole interactions, reviewed in (4, 5). This means that, on average, hydrophobic residues are present every 3.5 residues along the α-helix, slightly less than the 3.6 residues per α–helical turn, resulting in a super-helical twist along the coiled–coil axis (Fig. 1). A full 360° rotation of the coiled–coil is called a pitch, which varies locally but is mathematically optimal every ∼100 residues and spans over 150 Å (6). The exact pitch depends on the protein sequence of the hydrophobic core residues. Parameters that critically relate to the overall elastic properties of the coiled-coil and its ability to bend, and to dynamically adjust its shape in response to binding events and mechanical forces are the interhelical distance, which is the distance between the α–helical axes (7, 8), and local staggering which is the axial offset of individual residue pairs (7, 9).

**Figure 1:**
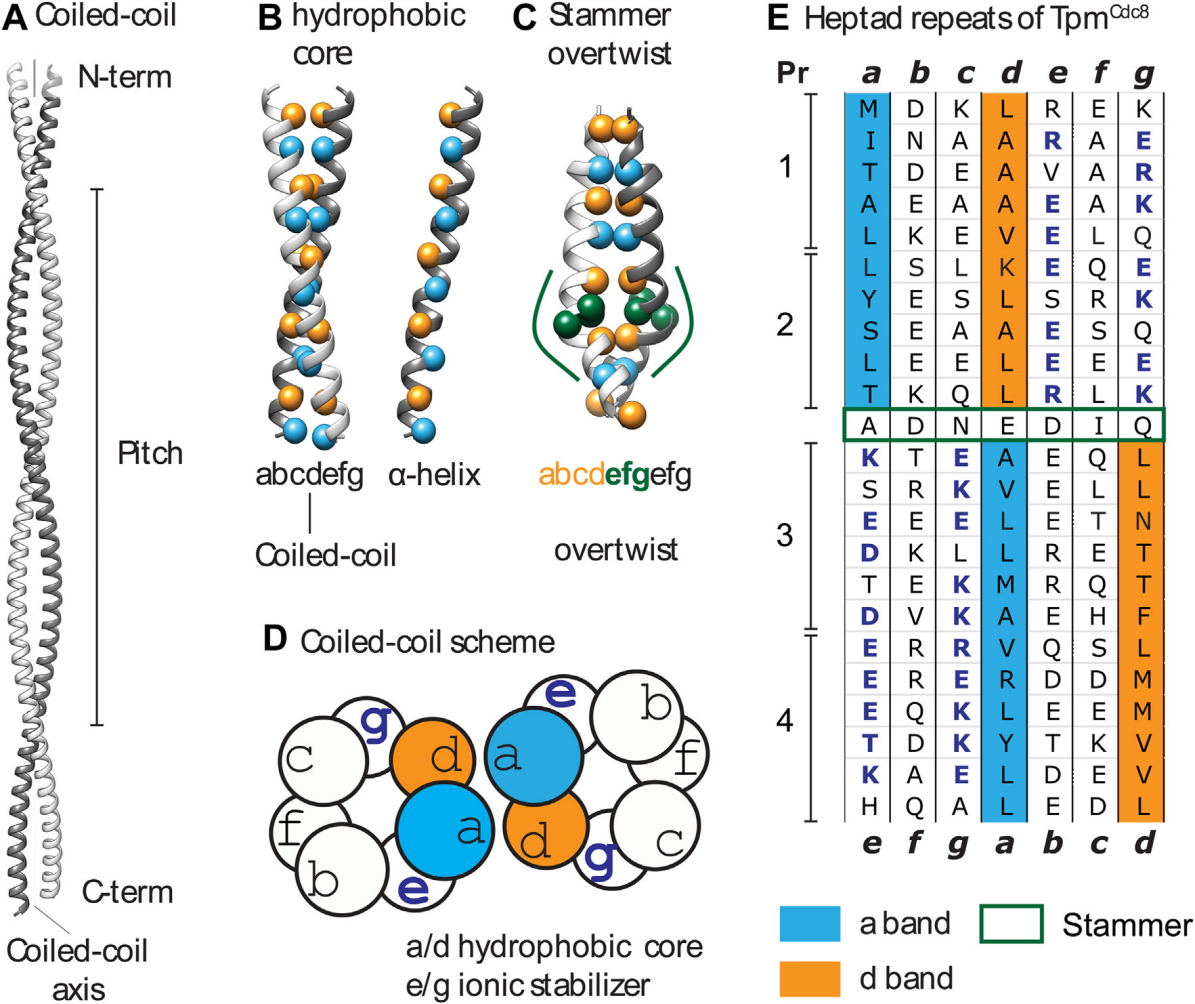
**Coiled–coil structure theory and Tpm**^**Cdc8**^**sequence.***A*, standard coiled-coil built with CC–builder 2.0 (56), an indication of N– and C–terminus, pitch, and coiled–coil axis. *Right*-handed α–helix twisting around the coiled–coil axis resulting in a left-handed coiled–coil. *B*, *left panel* shows coiled–coil with *blue* and *orange* balls indicating the *a-* and *d*-positions along the coiled–coil. Helix orientation is the same as in panel *A*. The *right panel* shows only one helix. *C*, coiled–coil example of a stammer (PDB: 1LR1) (83), depicting the addition of three residues in *green*. *Green lines* indicate overtwist. *D*, scheme of the heptad repeat interactions of coiled–coils. Indication of hydrophobic core bands and ionic stabilizer. *E*, Tpm^Cdc8^ contains four pseudorepeats (Pr), each consisting of up to six heptad repeats. Heptad repeat sequences with the hydrophobic core residues in the *a*-band (*cyan*) and *d*-band (*orange*) highlighted. The stammer, which induces a band shift, is marked by a *green box*. Ionic stabilizing residues on *e*– and *g*–position are shown in *blue bold*.

In fungi and metazoa, the members of the Tpm family regulate numerous and diverse functions of filamentous actin (F–actin) by controlling the dynamics, separation, and branching of the filaments as well as the access of actin-binding proteins such as cofilin, fimbrin and myosin to the surface of the filaments (10, 11, 12). In vertebrates, all sarcomeric F–actin and most of the cytoskeletal F–actin are present in the form of co-filaments with Tpm isoforms (9, 13, 14). The genes encoding vertebrate Tpm isoforms are referred to as TPM1 to TPM4 (15). In the sarcomere, the reversible azimuthal repositioning of muscle-specific Tpm isoforms on actin under the influence of troponin, myosin, and calcium ions plays a key role in the regulation of muscle contraction and relaxation (16, 17, 18). In contrast, cytoskeletal Tpm isoforms perform their various functions in the absence of troponin or isofunctional troponin–like proteins in the context of a much greater diversity of Tpm isoforms and by partitioning the various isoforms to functionally distinct actin filament populations (10, 19, 20, 21, 22). In mammals, cytoskeletal Tpm isoforms are subject to extensive qualitative and quantitative regulation between different tissues. The expression levels, modifications, interaction partners, and subcellular localization of specific Tpm isoforms differ significantly between various cell types and across developmental stages (23, 24, 25). Many cytoskeletal Tpm isoforms exhibit tissue-specific expression, contributing to the specialized function of the actin cytoskeleton in distinct tissues. This diversity allows Tpm isoforms to regulate actin filament stability and interactions in a context-dependent manner, tailored to the unique demands of different cell types and developmental phases (26).

Structural studies of Tpm have been challenging due to its inherent flexibility and the high solvent content typically present in crystals. Initial insights into Tpm's structure came from paracrystals of rabbit smooth muscle tropomyosin, first reported in 1966 (27, 28). These orthorhombic paracrystals revealed Tpm oriented diagonally, with a length of 400 Å, providing an early estimate of the length of a single Tpm coiled-coil. These studies suggested that Tpm adopts a supercoiled structure, and it was observed that the crystals shrank upon dehydration — an indication that Tpm dimers are interconnected, suggesting early evidence of dimer linkages. The diagonal packing of Tpm in these para crystals differs markedly from the unit cell structure observed in our Tpm^Cdc8^ crystals, highlighting the variability in crystal packing and arrangement between different Tpm isoforms and experimental conditions. This difference underscores the complexity of studying Tpm's structure across different contexts (see Fig. S1). A high-resolution full-length structure has yet to be determined for any member of the Tpm family, leaving fundamental questions unanswered. Nevertheless, low resolution full–length structures, molecular dynamic simulations as well as high-resolution NMR and X–ray crystallography studies of Tpm fragments (9, 13, 14, 29, 30) have confirmed key predictions from sequence analysis models. In particular, they have shown how core sequence anomalies and other deviations from an ideal heptad repeat structure help to break the rigid nature of a perfect coiled-coil structure. Adaptations, including the presence of alanine clusters, insertions, and the replacement of hydrophobic core residues with acidic residues, provide the Tpm isoforms with the conformational flexibility to associate with actin filaments (24, 31, 32, 33, 34).

In the absence of comprehensive structural information, the combination of sequence-based modeling with biochemical, molecular genetic, and cell biological approaches has significantly advanced our understanding of Tpm structure-function relationships (35, 36, 37). Electrostatic forces were shown to be primarily responsible for stabilizing the interaction between actin and Tpm in co-filaments (38). In addition, isoform-specific differences in sequence and posttranslational modifications in either the actin or Tpm protein impact key properties of co-filaments and thereby affect interactions with specific binding partners and modulate cellular functions (39). A common posttranslational modification is the N–terminal acetylation of Tpm. This posttranslational modification is present throughout all Tpm isoforms and has a regulating impact on Tpm–function. N–N-terminal acetylation was shown to alter the interaction with F–actin resulting in changes in binding affinity and interaction of the actin–Tpm filament with myosin (22, 40, 41). Moreover, it was shown that it affects the interaction between Tpm and tropomodulin (42).

Due to the simple composition of its actin-based cytoskeleton, the fission yeast, *Schizosaccharomyces pombe (S. pombe)*, has proven to be an attractive model organism for studying the function and regulation of cytoskeletal structures (43, 44). Most classes of actin-binding and actin-regulating proteins are present in *S. pombe*, but with fewer isoforms when compared to vertebrates. Thus, *S. pombe* produces one actin isoform, five myosin isoforms, three formin isoforms, and a single Tpm isoform. These components form three distinct types of actin-based structures in vegetative cells: actin cables, contractile rings, and actin patches (45), with Tpm^Cdc8^ localizing to actin cables and the cytokinetic contractile ring (38, 43, 46). Amino-terminal acetylation of Tpm^Cdc8^ increases its F–actin affinity six-fold and changes its cellular localization, revealing an alternative mechanism for functional diversification (41, 43, 44, 47, 48). N-terminal acetylation of Tpm^Cdc8^ represents a direct regulatory mechanism that influences myosin function in a class-dependent manner in yeast cells (48). It has been suggested that acetylation of Tpm^Cdc8^ acts as a sorting signal for the generation of distinct actin populations in a formin–directed manner (49). However, selective incorporation of acetylated Tpm^Cdc8^ into filaments being solely due to direct physical interaction with full-length formins has yet to be established (50). Different from the vertebrate Tpm isoforms, Tpm^Cdc8^ has a central stammer, the deletion of four residues in a central heptad repeat (Fig. 1, *C* and *E*) (8, 43). The presence of a single alanine cluster near its N-terminus supports the hypothesis, derived from comparing Tpm sequences across different species, that core alanine clusters are more conserved only in those that bind troponin.

Here, we describe the first high–resolution crystal structure of acetylated and unacetylated Tpm^Cdc8^. We have solved the structures of three full–length unacetylated Tpm^Cdc8^ conformers (conf-U1, conf-U2, and conf-U3) and one acetylated Tpm^Cdc8^ conformer (conf-A1) with a resolution of 2.2 (conf-U1 & A1) to 2.4 Å (conf-U2 & U3). The homodimeric structures form long cables, where the N-terminus of one homodimer forms overlapping head–to–tail contacts with the C-terminus of a neighboring homodimer. The implications of our results shed new light on the structure of the central stammer and the overlap junction. Moreover, obtaining the structures of both the unmodified and acetylated protein enables accurate determination of structural changes induced by N-terminal acetylation.

## Results

### Crystal structures of Tpm^Cdc8^ cables

**Figure 2:**
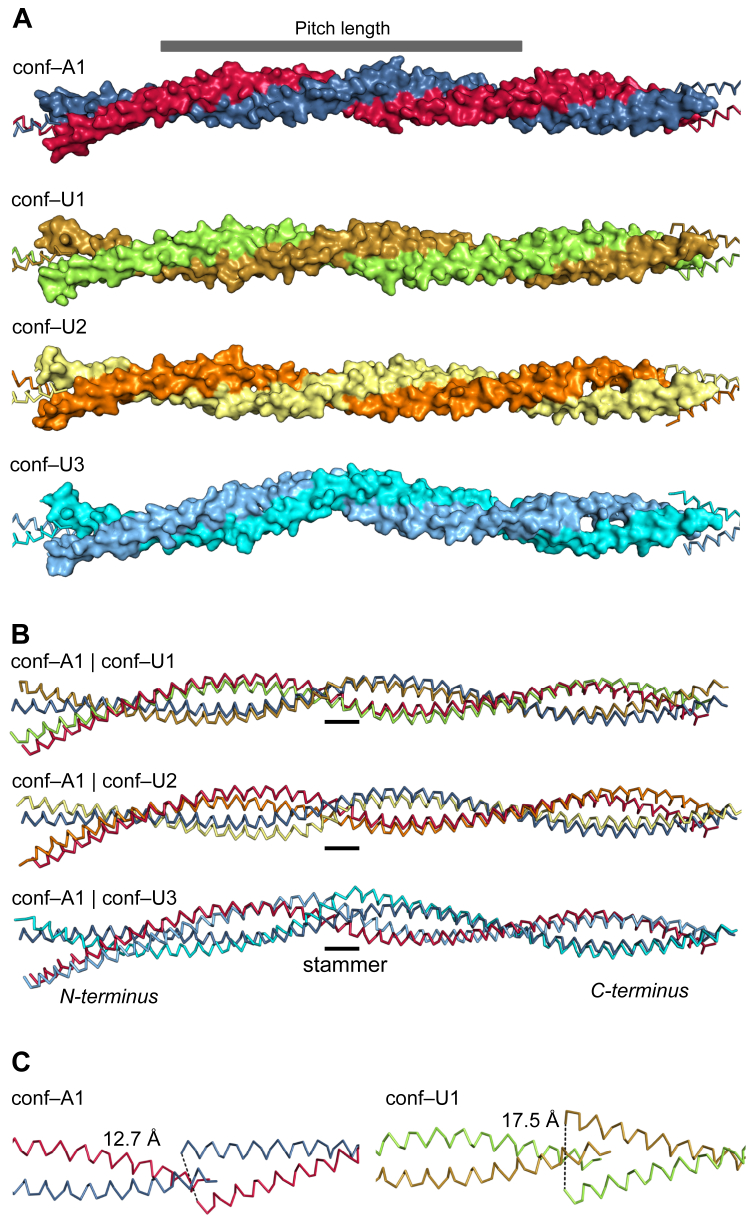
**Crystal structures of Tpm**^**Cdc8**^**dimers and their overlap junctions.***A*, surface representation of the four Tpm^Cdc8^ cable structures, conf-A1 (chain A, C – *blue*, chain B, D – *red*), conf-U1 (chain A, C – *gold*, chain B, D – *green*), conf-U2 (chain A, C – *yellow*, chain B, D – *orange*), conf-U3 (chain A, C - *blue*, chain B, D – *light blue*). The pitch length (89.4 residues, 130 Å) is depicted at the *top*. *B*, overlay of Tpm^Cdc8^ conf-A1 (*blue*, *red*) and conf-U1 (*gold*, *green*), conf-A1 (*blue*, *red*) and conf-U2 (*yellow*, *orange*) and conf-A1 (*blue*, *red*) and conf-U3 (*blue*, *light blue*) the stammer region is indicated by a bar. *C*, overlap junction of conf-A1 (*left panel*) and conf-U1 (*right panel*), with the C–terminal part of the coiled–coil on the *left* and the N–terminal part of the coiled-coil on the *right*. The distance between the C-alpha atoms of the AcM1 or M1 residues in the chains that form a Tpm^Cdc8^ dimer, representing the width of the overlap junction, is depicted as a *dashed line*.

High-resolution structures of full-length Tpm^Cdc8^ were obtained by X-ray crystallographic analysis. Single crystals of acetylated, unacetylated native, and SeMet–derivatized Tpm^Cdc8^ were grown at 18 °C by vapor diffusion using the sitting-drop method. The SeMet–derivatized unacetylated Tpm^Cdc8^ crystallized in space group P2_1_ and diffracted to 2.4 Å resolution (Table 1). The structure was solved by Single wavelength Anomalous Dispersion (SAD) phasing. Here, the asymmetric unit contains two Tpm^Cdc8^ homodimers of different conformations (Fig. 2*A*; conf-U2 and conf-U3). Native, unacetylated Tpm^Cdc8^ yielded crystals in space group P1 with one homodimer in the asymmetric unit and a diffraction limit of 2.2 Å (Fig. 2*A*; conf-U1). Most likely, these three different conformers represent trapped states in response to different elastic and torsional loads. Conf-U1 and conf-U2 have a straight coiled–coil structure, whereas conf-U3 contains a central 24° kink. All homodimer conformers are approximately 230 Å in length and exhibit head–to–tail interactions, where the N–termini of one homodimer contact the C–termini of an adjacent homodimer. The unit cell contains segments of multiple Tpm^Cdc8^ molecules, collectively encompassing the entire dimer structure (exemplary depicted in Fig. S1 for conf-A1). The overlap junctions connecting the Tpm^Cdc8^ homodimers with each other to form long Tpm^Cdc8^ cables exhibit discrete differences.

**Table 1:** Crystallographic data, phasing, and refinement statistics

Protein	Tpm^Cdc8^ acetylated	Tpm^Cdc8^ unacetylated	Tpm^Cdc8^ SeMet derivative
Conformer	A1	U1	U2, U3
PDB–code	9FF9	8PUZ	8PV0
Data collection			
Beamline	I03, Diamond	BM30A, ESRF	P13, DESY/PETRA–III
Space group	P1	P 1	P 2_1_
Cell parameters: *a*, *b*, *c* [Å] *α, β, γ* [º]	25.4, 38.3, 97.5 97.7, 94.7, 101.5	23.3, 38.6, 98.9 94.3, 91.9, 102.9	46.5, 77.7, 108.2 90, 94.2, 90
Wavelength [Å]	0.97627	∼0.98	0.9795
Resolution range [Å]	37.1–2.2 (2.5–2.2)^a^	24.6–2.2 (2.3–2.2)	107.7–2.4 (2.5–2.4)
Completeness [%]	78.0 (51.3)^b^	99.9 (99.8)	98.9 (98.3)
Multiplicity	3.3 (2.6)	13.0 (12.2)	6.0 (6.0)
<I/σ(I)>	8.0 (2.2)	10.5 (2.3)	9.8 (1.9)
R_merge_ [%]	6.8 (51.5)	13.3 (70.3)	8.8 (55.5)
CC_1/2_	0.998 (0.835)	0.999 (0.915)	0.999 (0.887)
Ellipsoidal resolution (Å) (direction)	2.976 (0.344 a∗ + 0.155 b∗ − 0.926 c∗) 2.087 (−0.247 a∗ + 0.467 b∗ − 0.849 c∗) 3.633 (0.031 a∗ + 0.360 b∗ + 0.933 c∗)		
Phasing statistics			
Number of sites			16
Phasing power			2.53
R_Cullis_			0.88
Overall figure of merit (observed/after density modification)			0.500/0.642
Refinement statistics			
Number of protein chains in a.u.	2	2	4
Included amino acids for each chain	1–161	1–161	1–161
No. of protein atoms	2658	2652	5304
No. of waters	19	35	64
Matthews coefficient	2.5	2.4	2.7
Sovent content	51.4%	48.4%	54.3%
R_work_/R_free_ [%]	26.1/31.4	23.3/28.0	25.1/31.1
r.m.s.d. for bonds [Å]/angles [°]	0.002/0.34	0.002/0.32	0.002/0.38
Ramachandran favored/allowed [%]/outliers [%]	100.0/0.0/0.0	100.0/0.0/0.0	99.5/0.5/0.0
Average B–factor macromolecule [Å^2^]	45.72	63.06	80.00
Average B–factor water [Å^2^]	23.33	64.36	68.61

a Values in parentheses are for the highest–resolution shell.

b Ellipsoidal completeness.

Despite the high similarity of space group and unit cell parameters with the structure of the unacetylated protein in space group P1 (Table 1), solving the structure of acetylated Tpm^Cdc8^ by molecular replacement using regions of the unacetylated structure as search models repeatedly failed. We were able to solve this structure by molecular replacement of ideal helical fragments using the coiled-coil mode of ARCIMBOLDO-LITE (51). The crystals diffracted to a resolution of 2.2 Å; however, completeness significantly declined beyond 3 Å due to anisotropic effects (Fig. S2). Isotropic processing yielded 82% completeness with a high-resolution cut-off of 2.5 Å. After accounting for anisotropy, the effective resolution of conf-A1 was approximately 2.4 Å. We consider the anisotropic processing method to be appropriate and effective in preserving high-quality data, as evidenced by the electron density map.

### The coiled–coil structure of Tpm^Cdc8^ cables

Each of the conformers that make up the Tpm^Cdc8^ cables consists of two α-helices that are wrapped around each other, forming a left-handed coiled–coil structure. In all four conformers, T15 is the first hydrophobic core residue in the a-position to interact with the dimer counterpart. In each case, the two chains wind around each other approximately every 45 residues, corresponding to 63 to 65 Å (Fig. 2*A*). Thus, the 161 residues of Tpm^Cdc8^ span 1.8 helical pitches, with the pitch length defined in Fig. 1*A*, equating to about 130 Å or 89 to 90 residues per pitch. By comparison, the high-molecular-weight mammalian isoform Tpm1.1, which comprises 284 residues, spans 2.75 pitches, equivalent to approximately 143 Å or 103.3 residues per pitch (52, 53). The interhelical radius of Tpm^Cdc8^ varies between 4 and 5.5 Å, consistent with previous observations reported for mammalian Tpm structures (46, 53, 54, 55). Different from structural models predicted by CCBuilder 2.0 or AlphaFold 3 (56, 57), the A and B chains of all four conformers display significant conformational differences and resulting asymmetry in their coiled-coil structures (Fig. 2*B*). This asymmetry can be seen by superimposing the Cα atoms of chain A and chain B of the same conformer and calculating the rmsd-value, which is 0 Å for the perfectly symmetric coiled–coil models predicted by CCBuilder 2.0. The rmsds for conf-A1, conf-U1, conf-U2 and conf-U3 are 5.9 Å, 3.5 Å, 4.4 Å and 9.0 Å, respectively. The Cα rmsd values between all chains range from 1.7 Å to 9.0 Å (Table 2). The largest difference between chain A and chain B of a given conformer is 9.0 Å for conf-U3, which in this case is primarily attributable to its central kink. The greatest similarity is shared by conf-U1 chain B and conf-U2 chain A with an rmsd of 1.7 Å. The most significant differences generally occur in regions likely to exhibit the highest conformational flexibility, specifically near the N-terminus, C-terminus, and the stammer region (Fig. S3). These regions likewise exhibit the greatest deviations from ideal coiled-coil geometry, resulting in variations in interhelical distance, local curvature and pitch length (Fig. S4). All conformers show increased local curvature and decreased pitch length within the stammer region, indicative of enhanced torsional and bending dynamics.

**Table 2:** Root mean square deviation (rmsd) between C–alpha atoms of all Tpm^Cdc8^ structures

Structure	Chain	Conf–A1		Conf–U1		Conf–U2		Conf–U3	
		A	B	A	B	A	B	A	B
conf–A1	A								
	B	5.9 Å							
conf–U1	A	2.9 Å	4.0 Å						
	B	3.9 Å	3.1 Å	3.5 Å					
conf–U2	A	4.0 Å	3.6 Å	3.6 Å	1.7 Å				
	B	3.5 Å	3.9 Å	2.1 Å	3.8 Å	4.4 Å			
conf–U3	A	4.6 Å	6.5 Å	5.7 Å	4.9 Å	4.3 Å	6.8 Å		
	B	7.4 Å	4.1 Å	5.6 Å	5.4 Å	5.7 Å	5.0 Å	9.0 Å	

Another key parameter for characterizing Tpm cable structures is the angle of twist of the overlap junction. This is the angle between two planes that extend between the two termini of each homodimer and its central coiled–coil axis. This angle varies among the observed structures, ranging from 75.5° in conf-U3 to 98.3° in conf-A1 (Table 3), demonstrating the significant flexibility of the overlap junction. In contrast to our observation, molecular dynamics studies have predicted more narrowly defined twist angles in the range from 85.7° to 90.6° for smooth and striated muscle Tpm isoforms, respectively (18).

**Table 3:** Twist angles, overlap area, number of contacts per area for all four structures

Structure	Overlap length^a^	Twist angle	Buried overlap area	Number of contacts in the overlap junction^b^	Number of contacts per 1000 Å^2^
conf–A1	5.8 Å	98.26°	1279.0 Å^2^	47	36.8
conf–U1	9.3 Å	80.20°	1531.1 Å^2^	33	21.6
conf–U2	12.5 Å	80.48°	1877.4 Å^2^	51	27.2
conf–U3	10.9 Å	75.54°	1379.8 Å^2^	31	22.5

a Distance between the C-terminal end of the coiled-coil and the N-terminal end of the coiled-coil.

b Contacts between the N- and C-terminus are defined as instances where the VDW overlap between two atoms, defined as the sum of their VDW radii minus the distance between their centers, is in the range −0.4 to 0.6 Å.

### Impact of N-terminal acetylation

**Figure 3:**
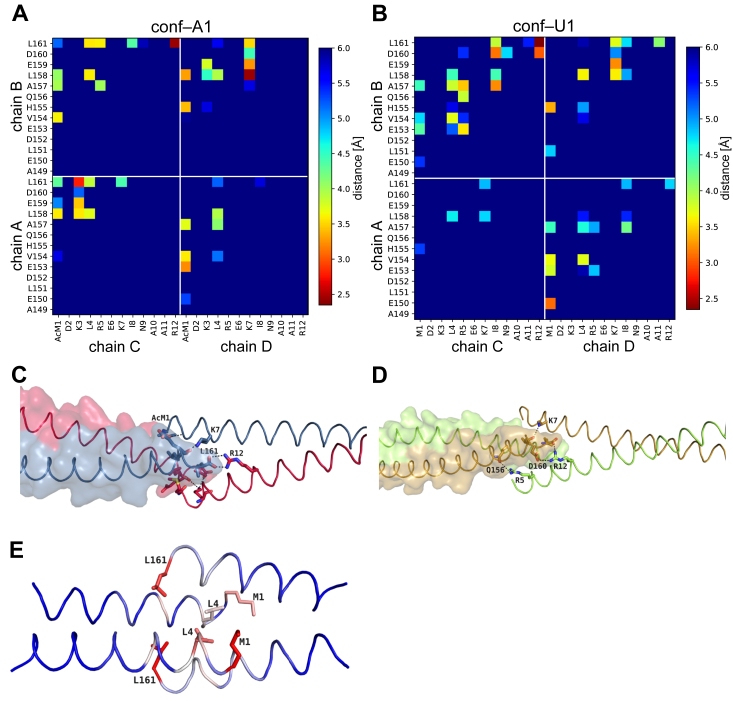
**Comparison of acetylated and unacetylated overlap junctions.***A*, distance map of the overlap junction of conf-U1. *B*, distance map of conf-A1. The distance maps show the minimal distances between the residues from the N-terminal part and the C-terminal part. Distances above 6 Å were cut–off. *C* and *D*, the overlap junction of conf-A1 and conf-U1, respectively, are shown in ribbon form to illustrate the stabilizing interactions. Side chains are shown for the last 12 residues. The coloring of the ribbons and of the surface of the N-terminal chains match the coloring in Fig. 2*A*. *E*, conf-U1 colored by distance change to C-terminal residues of the overlap junction compared to conf-A1, large changes (*red*), medium changes (*white*), small changes (*blue*).

To analyze the impact of N-terminal acetylation, we compared the acetylated structure (conf-A1) with conf-U1, which has the highest structural similarity (rmsd of 3.0 Å). N-terminal acetylation of Tpm^Cdc8^ induces structural changes that result in a different architecture of the overlap junction. The carbonyl oxygen atom of the acetyl group forms an additional hydrogen bond with the backbone nitrogen atom of residue L4 within the same helix, thereby stabilizing the helical structure at the N-terminus. Additionally, the methyl group of the acetyl group forms a hydrophobic interaction with the residues L158 and H155 of the C-terminal coiled-coil. A corresponding increase in helicity as a result of N-terminal acetylation has been reported for the N-terminal fragment of Tpm1.1 (9, 58). The increased helicity triggers a change in the overall architecture of the overlap junction, with the result that the acetylated N-termini, which are less flexible, move closer together. The distance between the methionine Cα atoms of chains A and B shortens from 17.5 Å in the unacetylated structure to 12.7 Å in the acetylated structure (Fig. 2, *B* and *C*). Hence, the interacting N– and C–terminal residues of this more compact overlap junction form more stable interactions. In contrast, the electron density for the N-terminal methionine residues in conf–U1 is not well defined, indicating greater flexibility within the N-termini. The shifts triggered by N–terminal acetylation translate into a changed interaction profile, which is visualized by a distance map of the two structures (Fig. 3, *A* and *B*). The distance maps reveal differences in the dimensions of the overlap junction. The overlap length for the acetylated conf–A1 is reduced (5.8 Å) compared to conf-U1 (9.3 Å), which is also reflected in different buried areas of conf-U1 (1531.1 Å^2^) and conf–A1 (1279.0 Å^2^) (Table 3). Moreover, the distance maps show that the overlap junction is not highly symmetrical with respect to the coiled-coil axis. Different interaction profiles between the chains can be observed for all chains (Figs. 3, *A* and *B* and S5). Key ionic interactions, stabilizing the overlap junction of conf-A1 include those between residue R12 and the C–terminus as well as residue K3, the C-terminus and residue E159 (Fig. 3*C*). Residue R12 of conf-U1 also interacts with the C-terminal part of the next coiled-coil, but here residue R12 interacts with the C-terminus and the side chain of D160. In conf–U1, the majority of the interactions stabilizing the overlap junction are of hydrophobic nature. In addition, residues K7 and E159 form a salt bridge (Fig. 3*D*). The quality of the electron density for the overlap junction differs between conf-U1 and conf–A1. There is good electron density for all amino acid side chains and even for the N-terminal acetyl group in conf–A1 (Fig. S6*A*), whereas in conf–U1, the electron density in the overlap junction is missing for some amino acid side chains (Fig. S6*B*).

### Stability of the overlap complex

**Figure 4:**
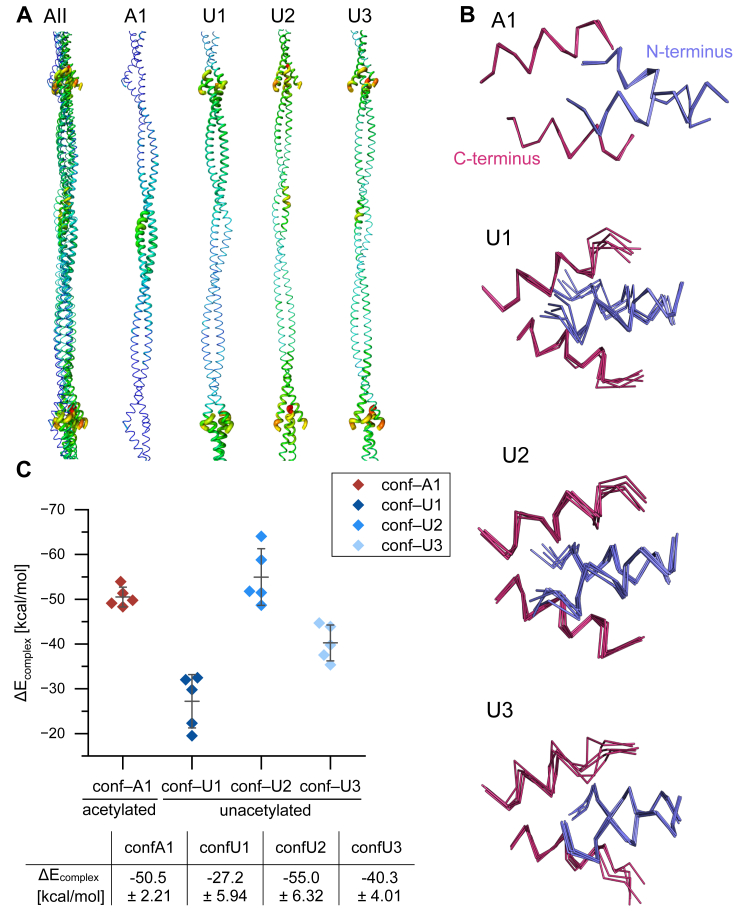
**Conformational flexibility and assembly energies of the overlap junction.***A*, B-factor analysis of all conformers (All) and the individual conformers (A1, U1, U2, U3), colored by B-factor. *B*, superposition of the five ensemble structures with the lowest R_free_ values. The C- and N-termini are colored red and blue, respectively. *C*, binding energy of the overlap junction. The energy values were calculated for the five ensemble structures with the lowest R_free_ values. The table summarizes the corresponding mean values of the energy changes (mean ± standard deviation).

To better understand the dynamics of the overlap junction interaction, we performed crystallographic ensemble refinement of the four conformers, resulting in ensemble structures representing the dynamics of the cable structures (Fig. S7), consistent with the experimental diffraction data. As predicted by an analysis of the B-factors of the conformer structures (Fig. 4*A*), conf A1 showed a narrow ensemble distribution, while those for conf-U1, conf-U2 and conf-U3 were much wider indicating increased flexibility of their overlap junctions (Fig. 4*B*). For conf-A1, higher flexibility was observed in the stammer region (Fig. 4*A*). We then calculated the assembly energy of the overlap junction using a molecular mechanics approach. The distribution of the resulting energies is consistent with the observed flexibility (Fig. 4*C*). The interaction energies of the conformers are −50.5 kcal/mol for conf-A1, −27.2 kcal/mol for conf-U1, −55.0 kcal/mol for conf-U2 and −40.3 kcal/mol for conf-U3. The acetylation therefore leads to a more energetically favorable overlap junction compared to conf-U1 and the average of all unacetylated conformers (−40.8 ± 12.79 kcal/mol). Aside from the effect of acetylation, we observe a broad range of energies for the different states in which the unacetylated Tpm^Cdc8^ is trapped.

### Implications for known Tpm^Cdc8^ variants

**Figure 5:**
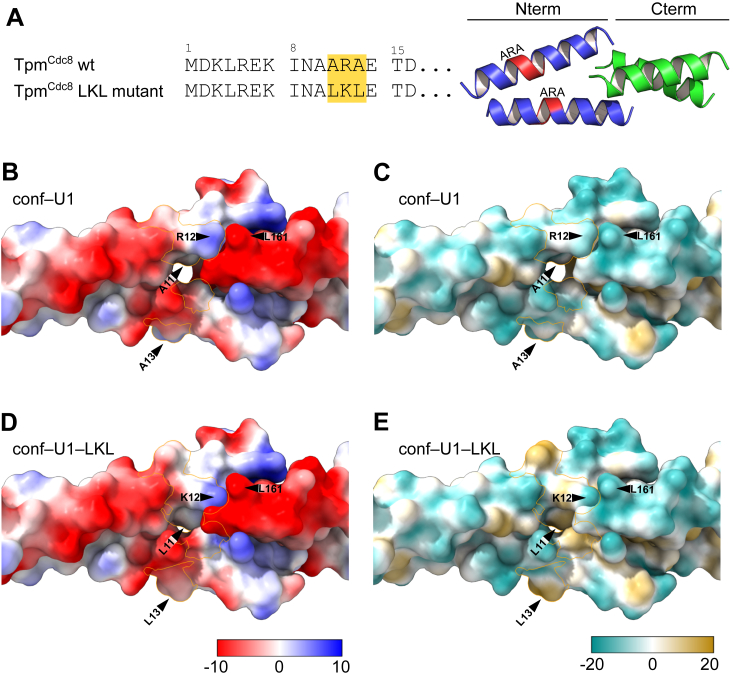
**Impact of the replacement of the -A-R-A- residues at positions 11 to 13 with the -L-K-L- motif on electrostatic and lipophilicity potentials.***A*, sequence alignment of N-terminal residues with residues 11 to 13 (ARA) highlighted (*left*) and ribbon representation of the 20 terminal amino acids of the overlap complex, C-terminus (*green*) N-terminus (*blue*) with the residues 11 to 13 highlighted in *red*. Surface representation of the overlap complex of conf-U1 (*B* and *C*) and conf-U1–LKL (*D* and *E*) with the mutated residues 11 to 13 highlighted by an *orange* border. *B*, surface is colored according to the electrostatic potential of conf-U1. *C*, surface is colored according to the lipophilicity potential of conf-U1. *D*, surface is colored according to the electrostatic potential of conf-U1–LKL. *E*, surface is colored according to the lipophilicity potential of conf-U1–LKL. Look-up tables for Coulombic electrostatic potential (range −10 to +10) and molecular lipophilicity potential (range −20 to +20) were used.

Replacement of the -A-R-A- residues at positions 11 to 13 with the -L-K-L- motif, which is conserved at the same position in the Tpm isoforms of mammalian muscle, has been shown to stabilize both the helical structure and dimerization of Tpm^Cdc8^. The triple mutant A11L/R12K/A13L shows an enhanced tendency for cable formation and a reduced affinity for actin (47). Our structures reveal that residue R12 forms salt–bridges with the C–terminus of an adjacent homodimer and the carboxylic acid side chain of residue D160 as well as hydrophobic interactions with residue L161. We mutated residues 11 to 13 to L–K–L *in silico* and analyzed the impact on the electrostatic and hydrophobic surface in this region (Fig. 5). Replacing A11 with L11 in the *d*-position introduces an additional hydrophobic core contact, which accounts for the increased dimer stability observed in (47). Conversely, substituting R12 disrupts the formation of a salt bridge with the C-terminal carboxyl group of L161 on one side of the coiled-coil (Fig. 5, *B* and *D*). Residue A13 in the *f*–position is exposed to the solvent. Mutating A13 to leucine enhances the hydrophobic surface area exposed to the solvent on both sides of the coiled-coil Overall, the presence of the A-R-A- residues appears to increase the conformational flexibility of the region, thereby improving the ability to modulate the physical properties of the sole Tpm isoform produced in *S. pombe*.

### Actin–Tpm^Cdc8^ co-filament model

**Figure 6:**
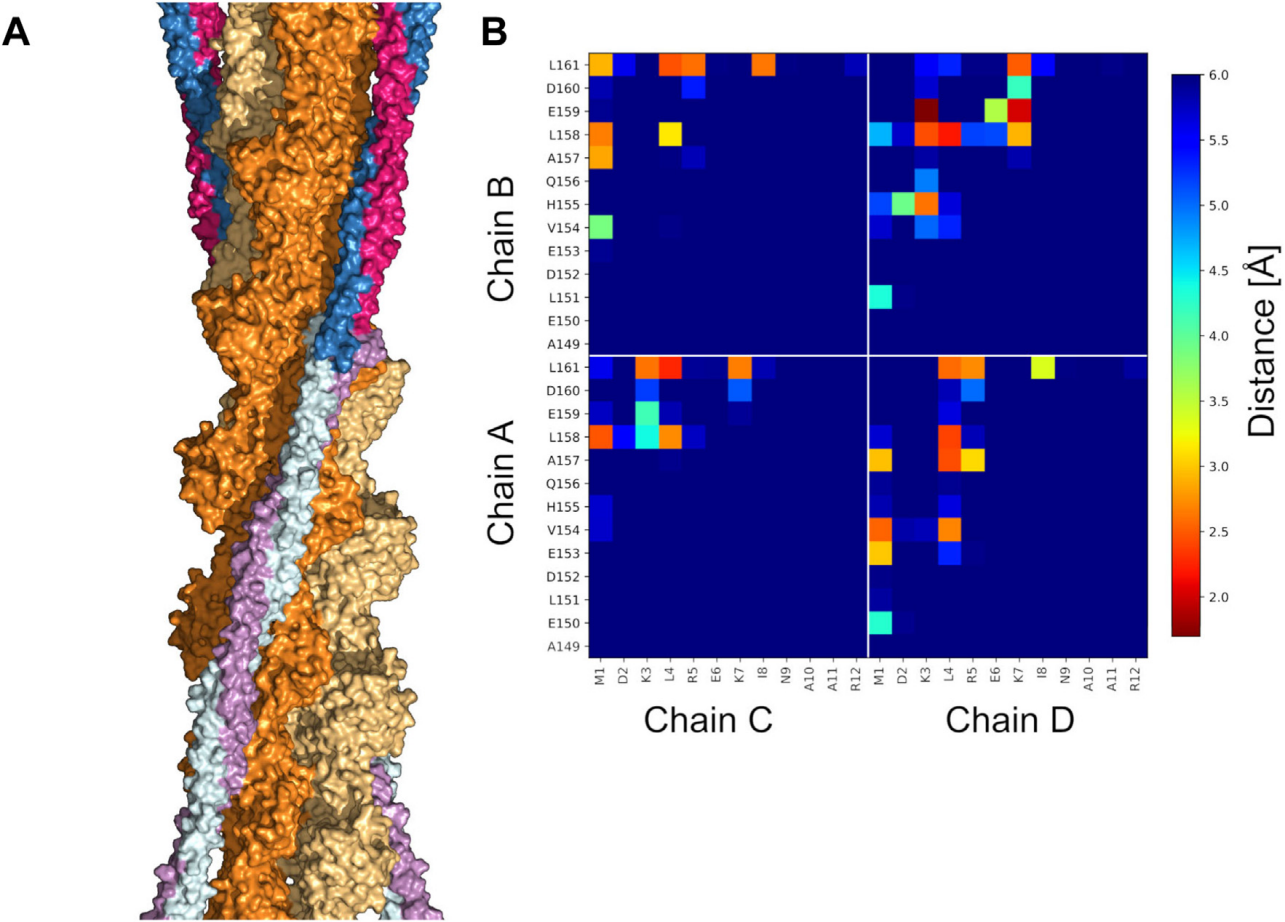
**Actin Tpm**^**Cdc8**^**co-filament model.***A*, ensemble of 10 conformations that fit the overall shape of the Tpm cable (extracted from 5JLF) obtained by computational modelling using conf-A1 as the starting structure. *B*, average distances between interchain contacts within the overlap junction for all 10 conformations. Distances are indicated in the range from 1.8 to 6 Å, using the "jet" color map for visualization.

The Tpm^Cdc8^ structures presented here lack the superhelical symmetry necessary for binding to the actin filament. To address this discrepancy, computational modeling was employed to position two coiled-coils of conf–A1 into the expected location of the Tpm cable along the actin filament (Fig. 6*A*). The modelled conformations of conf–A1 exhibited an alignment with the overall geometry observed in actin-bound Tpm complexes (Fig. 6*A*). The results indicate that, despite the absence of intrinsic superhelical symmetry in conf-A1, Tpm^Cdc8^ is sufficiently flexible to adopt conformations within the cable that are consistent with the binding mode of Tpm to the actin filament. Moreover, we measured the distances within the overlap junction (Fig. 6*B*) of the actin bound conf–A1, comparing them to the straight cables observed in our crystallographic data. The similarity in these distances indicates that conf-A1 is capable of adopting the superhelical structure crucial for actin binding, without significantly altering the geometry of the overlap junction. This similarity is also reflected in the low rmsd value between straight conf–A1 and the actin-bound conformer of only 0.542 Å for the overlap complex.

## Discussion

In this study, we present the crystal structures of native Tpm^Cdc8^ in its unacetylated and acetylated form and describe the differences between these structures and their implications. Previously reported Tpm structures were limited by their low resolution or by the fact that they only represented fragments of the protein (9, 13, 29, 59). Cryo-electron microscopy structures of actin-bound Tpm lack high-resolution information for the Tpm part and thus do not provide insights regarding conformational properties of the side chains and in particular the organization of the overlap junction. The elevated R-factors of our structures, ranging from 0.280 to 0.314, are likely attributable to pseudo-translational non-crystallographic symmetry (pseudo-tNCS), a common occurrence in structures with repeating coiled-coil motifs such as Tpm^Cdc8^. Pseudo-tNCS can modulate diffraction intensities, generating subtle off-origin peaks in the Patterson map. While meridional reflections at 5.1 Å, corresponding to the coiled-coil repeat, and equatorial reflections at 8 to 9 Å, indicative of inter-helical distances in dimeric coiled-coils, are clearly visible in the Patterson map, these peaks fall below the detection thresholds of standard tools like phenix.xtriage, which typically flags peaks only if they exceed 20% of the origin. As a result, pseudo-tNCS distorts diffraction data, causing unexpected intensity variations in reflection groups, akin to patterns seen in fibrous diffraction (60). The limitations of current refinement software in addressing these distortions contribute to the higher-than-average R-factors observed in such structures. For instance, a study by Thomas and colleagues reported an average R-free of 0.274 ± 0.0324 for 27 coiled-coil proteins, with values ranging from 0.170 to 0.316 (61). While the Tpm^Cdc8^ structures exhibit higher-than-average R_free_ values, they remain within the range observed for coiled-coil structures analyzed in this study. Similar elevations in R-factors have been reported for various coiled-coil structures, including the complex between the N-terminal region of SNAP25 and the SNARE region of syntaxin 1a (PDB ID: 1JTH), the N-terminal region of the scallop myosin rod (PDB ID: 3BAT), the human beta-myosin S2 fragment(PDB ID: 2FXO), and the mid-region of rat striated muscle α-tropomyosin residues 89 to 208 (PDB ID: 2B9C) (54, 62, 63, 64). These examples suggest that elevated R-factors are not uncommon in coiled-coil structures and can be attributed to inherent challenges in handling pseudo-tNCS effects, underscoring the need for more sophisticated refinement approaches to mitigate these distortions.

Structure prediction is an alternative way of obtaining structural information about the protein. In our case, the structure of an isolated Tpm^Cdc8^ dimer was predicted accurately by AlphaFold 3 (57) with a rmsd of only 1.56 Å when compared with conf-U1. The effect of N-terminal acetylation is not predictable because N-terminal acetylation is not one of the PTMs included in AlphaFold 3. Another unique feature of our Tpm^Cdc8^ structures is the presence of the overlap junction. AlphaFold 3 cannot predict a corresponding cable structure and therefore does not provide any information about the overlap junction. In the yeast *S. pombe*, N-terminal acetylation of Tpm^Cdc8^ functions as a sorting signal for actin filaments. In this context, acetylated Tpm^Cdc8^ acts as a signal for incorporation into the cytokinetic actomyosin ring (41, 48). The biochemical effect of acetylation of Tpm^Cdc8^ is to increase the stability of the overlap junction, resulting in increased actin affinity and persistence length of the Tpm^Cdc8^ cable (47). Taking these results into account, our findings are consistent with the notion that N-terminal acetylation promotes helix formation, thereby significantly limiting conformational flexibility at the overlap junction and promoting a strong interaction state. In contrast, the unacetylated terminal regions possess much greater flexibility, enabling the formation of overlap junctions that display greater variability in interaction strength.

The biophysical properties of several Tpm^Cdc8^ variants have been characterized *in vitro* (47, 65). Tpm^Cdc8^ produced by the temperature–sensitive Tpm^Cdc8^ mutant strain “110” (66) contains two mutations, A18T and E31K, with the former having a larger impact on the thermal stability of the protein *in vitro*. Alanine 18 is located within the flexible N-terminal alanine cluster. Based on our structural models, replacement with a threonine residue will increase local stiffness, which will directly affect dimer dynamics and chain pairing within the overlap junction, thus having a negative impact on actin binding. In contrast, the E31K substitution is predicted to have a minimal impact on the protein structure, due to its solvent-exposed position. These predictions are consistent with Tpm^Cdc8^ variants containing the A18T or E31K substitutions alone (65). The temperature-sensitive Tpm^Cdc8^ variant “27” (66) contains single point mutation E129K that causes an approximately 3-fold reduction in actin binding capacity compared with the wild-type protein. Residue E129 is located in the “*g*” position of the heptad repeat and forms a salt bridge with the heptad core breaking residue R130. The replacement of this negatively charged glutamate residue with a positive lysine abolishes this interaction. Reorientation of the lysine residue towards residues D132/E134 alters the conformation of this broken core region, resulting in a decreased dimerization capacity of Tpm^Cdc8^ and disruption of actin interaction contacts. Mutation D2A was reported to greatly reduce actin affinity (47). Its impact appears to be mainly associated with increased cleavage of the starting methionine, which in turn leads to the lack of N-terminal processing of Tpm^Cdc8^. This results in greater flexibility and a lower interaction strength at the overlap junction. In all conformer structures, the side chain of D2 points outward, implying that it does not significantly contribute to shaping the stability of the region. Additionally, residue D2 does not engage in specific or close contact within the overlap junction (Fig. S5).

Besides the contacts formed by the overlap junction, multiple other crystal contacts are present. These contacts occur due to the lateral stacking of Tpm^Cdc8^ dimers inside the unit cell (Fig. S1). Amino acids that show these contacts are spread evenly across the molecule and are not very prominent at the terminal ends of the coiled-coil (Fig. S8).

Our results overcome the limitations imposed by high-resolution structural bottlenecks, paving the way for a thorough and detailed analysis of the function-structure relationships of Tpm within the *S. pombe* system.

## Experimental procedures

### Protein production and purification

Production of recombinant *S. pombe* (Tpm^Cdc8^ (UniProt: Q02088 TPM_SCHPO) in *E.coli* was performed as described previously (41, 67). In the case of the SeMet-labeled protein, we used an auto–inducing culture medium (68). Acetylated Tpm^Cdc8^ was produced by co-expression with the N–terminal acetylation complex NatB (67). Protein purification was performed using fractionated heat denaturation followed by isoelectric precipitation at pH 4.55, resuspension in 5 mM Tris–HCl pH 7.0, and ion exchange chromatography (41).

### Crystallization and structure determination of Tpm^Cdc8^

Crystals were grown at 18 °C by vapor diffusion by mixing 8 mg/ml (452 μM) Tpm^Cdc8^ with an equal volume of reservoir solution in the sitting drop setup. The best diffracting crystals of the SeMet-labeled protein were obtained in 100 mM Tris–HCl pH 7.8, 0.15 M ammonium acetate, and 40% MPD. The unlabeled protein crystallized in 100 mM Tris–HCl pH 8.2, and 45% MPD. Crystals grew within 3 days. Harvested crystals were flash–frozen in mother liquor without additional cryo–protection.

Crystallographic diffraction data for the non-derivatized Tpm^Cdc8^ were collected at the ESRF synchrotron beamline BM30A (Grenoble), using ADSC Quantum 315r detector and the wavelength 0.9797 Å. The crystals of SeMet Tpm^Cdc8^ crystals were measured at the DESY/PETRA–III synchrotron beamline P13, using a PILATUS 6M–F detector and a similar wavelength of 0.9795 Å. Data sets were integrated with the XDS program suite (69). Scaling and merging of the datasets were performed using SADABS and XPREP programs (70). The final high–resolution diffraction limits were 2.2 Å for the non–derivatized and 2.4 Å for the SeMet crystals (see Table 1 for details).

The initial phasing data were obtained from the SeMet crystals using the Single wavelength Anomalous Dispersion (SAD) method. The SeMet derivative diffraction data collected at the selenium K absorption edge allowed us to identify 16 selenium sites in the a.u. with phasing power and R_Cullis_ of 2.53 and 0.88, respectively. These sites produced an initial set of phases with an overall figure of merit of 50%, which increased to 64.2% after density modification using the CCP4 suite and the parrot algorithm (71). The resulting electron density revealed substantial parts of the α-helical structure for four chains of Tpm^Cdc8^, which were built into the density (72) and used as a partial model for further phase improvement. The model was refined with PHENIX (73). We used the TLS option in PHENIX with the default setting of 1. Increasing the number of TLS segments had a slight negative effect. Morphing with PHENIX improved the fit to the unbiased electron density map in reciprocal space. After several refinement cycles, sidechain densities started to emerge. These were initially filled with glutamates, one of the most abundant amino acids in Tpm^Cdc8^. Upon completion of the long coiled-coil segments, the quality of the electron density for the side chains improved sufficiently to accurately position the extended side chains of lysine and arginine. The large aromatic side chains and seleno-methionine residues were used as reference points for sequence assignment. The Hendrickson–Lattman coefficients (74), in combination with the density modification procedure, were used during refinement cycles to increase signal–to–noise ratio and quality of electron density maps and decrease the effects of model bias. The process of model building and phase improvement was repeated until all four chains of Tpm^Cdc8^ in the asymmetric unit were completed and refined.

The non-derivatized structure of Tpm^Cdc8^ was determined by the Molecular Replacement method using the structures of SeMet conformers 2 and 3 as search models. In order to increase the signal–to–noise ratio for solutions of the rotation function, the molecular replacement search was performed with an asymmetric fragment of Tpm^Cdc8^ including the residues S50-E117 of chain A and E59-H118 of chain B. To account for the conformational flexibility of Tpm^Cdc8^, Normal Mode Analysis was employed to generate conformational intermediates of conformers 2 and 3. The angular sampling for the rotation function was set to < 1°. A search for the translation function was not necessary in this case due to the properties of the triclinic lattice. This strategy allowed us to obtain a well–contrasted solution which, after initial refinement by simulated annealing, was used as the starting coordinates for the ARP/wARP model (75) building and density improvement procedures. The resulting ARP/wARP density was of good quality and allowed the building of a complete model of the Tpm^Cdc8^ homodimer (conformer 1). All models have good quality and stereochemistry with no Ramachandran outliers and low rmsd values for molecular bonds and angles (see Table 1 for details).

Acetylated Tpm^Cdc8^ was crystallized at 6 mg/ml in 100mM Tris–HCl pH8.0, 0.35M ammonium acetate and 43% MPD. Crystals grew within 3 days in a 48–well, sitting drop, vapor diffusion plate at 18 °C. The diffraction data was collected at Diamond Light Source (Beamline I03) and processed using the autoPROC STARANISO pipeline (76) on the IspyB laboratory information management system. STARANISO typically results in lower reported completeness compared to traditional methods, but the retained data are of higher quality, which is more beneficial for accurate model refinement. The structure was solved by molecular replacement using the program ARCIMBOLDO_LITE, followed by refinement with PHENIX and Coot (51, 72, 77, 78).

### Coiled–coil architecture analysis

The coiled–coil architecture features of Tpm^Cdc8^ conformers were analyzed using TWISTER (https://pharm.kuleuven.be/apps/biocryst/twister.php; used August 2019, May 2024, (7). This algorithm was used for the generation of the coiled–coil parameter of interhelical distance, local pitch length, and local curvature.

Rmsd values, contacts and distances were calculated using ChimeraX (79), and python scripts making use of the library MDAnalysis (80).

### Overlap complex analysis

In order to characterize the geometry of Tpm overlap junctions, two parameters were defined by (18). The first parameter, omega (ω), describes the linearity of both chains and determines the grade of bending along the overlap junction. The twist angle theta (θ) was measured by the angle between the triangular planes r1(P1, P2, P3) and r2(P4, P5, P6). Plane r1 is defined by the points P1, P2, and P3, where P1 and P2 represent the center of mass coordinates of residues 157 to 159 of the C-terminal chains, respectively. Point P3 is located at the center of the coiled-coil, defined by the center of mass of residue 147 from both C-terminal chains. Similarly, plane r2 is defined by points P4, P5, and P6, where P4 and P5 correspond to the center of mass coordinates of residues 4 to 6, and P6 is the center of mass of residue 14 from both chains.

The buried interface area was calculated by subtracting the solvent-accessible surfaces (SAS) of two isolated from two connected dimers using the “measure buriedarea” function of ChimeraX 1.8 (79).

### Generation of ensemble structures and calculation of complex formation free energy

The ensemble structures of all conformers were generated using the ensemble refinement method of the PHENIX suite (78). The five structures with the lowest R_free_ values were selected from the resulting ensembles and these were truncated to the last 12 N- and C-terminal residues. Molecular mechanics calculations were performed in HyperChem 8.0 using the MM + force field. Prior to the free energy calculations, a geometric optimization was carried out, with the protein backbone being constrained. The energies for the N-terminal part, the C-terminal part and the entire overlap junction were calculated separately and the free energy gain was calculated by subtracting the sum of the energies for the free terminal parts from the energy of the overlap junction.

### Generation of an actin-Tpm^Cdc8^ co-filament model

Two coiled-coil molecules of conf–A1 were subjected to molecular dynamics simulations in explicit solvent using the GROMACS simulation package with the CHARMM36 force field (81, 82). The protein was protonated and positioned within a rectangular box, with a minimum distance of 2 nm between the protein and the box walls. The box was solvated and sodium chloride was added to a final concentration of 0.15 M, thereby balancing the overall charge of the system to zero. Subsequently, the system was energy minimized. NVT and NPT equilibration was conducted for 100 ps, using the Parrinello-Rahman barostat in the latter case. The production simulation was done for 700 ns at 300 K using the V-rescale thermostat, with the pressure maintained at 1 bar through the use of the Parrinello-Rahman barostat. Long-range electrostatic interactions were treated with the Particle Mesh Ewald (PME) method, with a cutoff of 1.2 nm.

The density attributable to tropomyosin was extracted from the actin-Tpm structure (PDB: 5JLF, EMD-8162) and every 10th frame was fitted into this map using ChimeraX (79). The relevant fits were included in the subsequent analysis.

## Data availability

All reported structures can be found in the Protein Databank (PDB) under the following pdb codes: conf-U1: 8PUZ, conf-U2 and conf-U3: 8PV0 and conf-A1: 9FF9.

## Supporting information

This article contains supporting information.

## Conflict of interest

The authors declare that they have no conflicts of interest with the contents of this article.
