# Supplementary material for: Crystal structures of cables formed by the acetylated and unacetylated forms of the Schizosaccharomyces pombe tropomyosin ortholog TpmCdc8
Source: J Biol Chem. 2024 Oct 25;300(12):107925. doi: 10.1016/j.jbc.2024.107925 (PMC11626781; doi:10.1016/j.jbc.2024.107925)
Supplement: Supporting information [file mmc1.pdf]

Supporting information

Crystal structures of cables formed by the acetylated and unacetylated forms of the *Schizosaccharomyces pombe* tropomyosin orthologue Tpm^{Cdc8}

Patrick Y.A. Reinke[1,2,3,†], Robin S. Heiringhoff[1,2,†], Theresia Reindl[1, §], Karen Baker[4], Manuel H. Taft[1], Alke Meents[3], Daniel P. Mulvihill[4], Owen R. Davies[5], Roman Fedorov[1,2], Michael Zahn[1,2] and Dietmar J. Manstein[1,2,*]

From the [1] Institute for Biophysical Chemistry, Fritz–Hartmann–Centre for Medical Research, Hannover Medical School, 30625 Hannover, Germany, [2] Division for Structural Biochemistry, Hannover Medical School, 30625 Hannover, Germany, [3]FS–BMX, Deutsches Elektronen–Synchrotron DESY, Notkestraße 85, 22607 Hamburg, Germany, [4] School of Biosciences, University of Kent, Canterbury, Kent CT2 7NJ, UK,
[5] Wellcome Centre for Cell Biology, Institute of Cell Biology, University of Edinburgh, Michael Swann Building, Max Born Crescent, Edinburgh, UK,

[§] Current address: Department of Microbiology and Immunology, Stanford University School of Medicine, 279 Campus Drive West, Stanford CA 94305, USA

* Corresponding author: Dietmar J. Manstein, Tel: (49) 511–5323700; Fax: (49) 511–5325966; E–mail: manstein.dietmar@mh–hannover.de

†These authors contributed equally to this work

Supplementary Figures

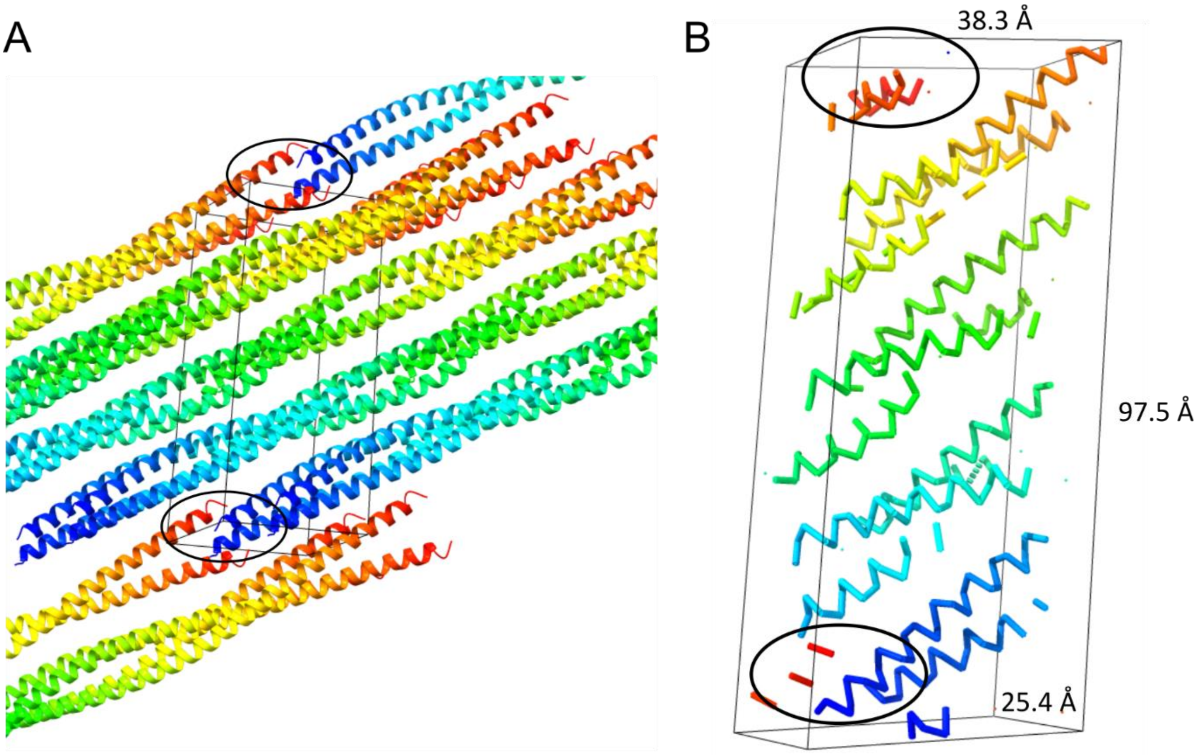

Supplementary Figure 1. Unit cell parameters for conf–A1. *A*, Symmetry-related variants of the conf-A1 structure covering the unit cell, color-coded from the N-terminus (blue) to the C-terminus (red) using a rainbow gradient. *B*, The unit cell of conf-A1 with residues contained therein. The rainbow gradient matches the coloring from panel A. The location of the overlap junction is marked by ellipsoids for clearer identification.

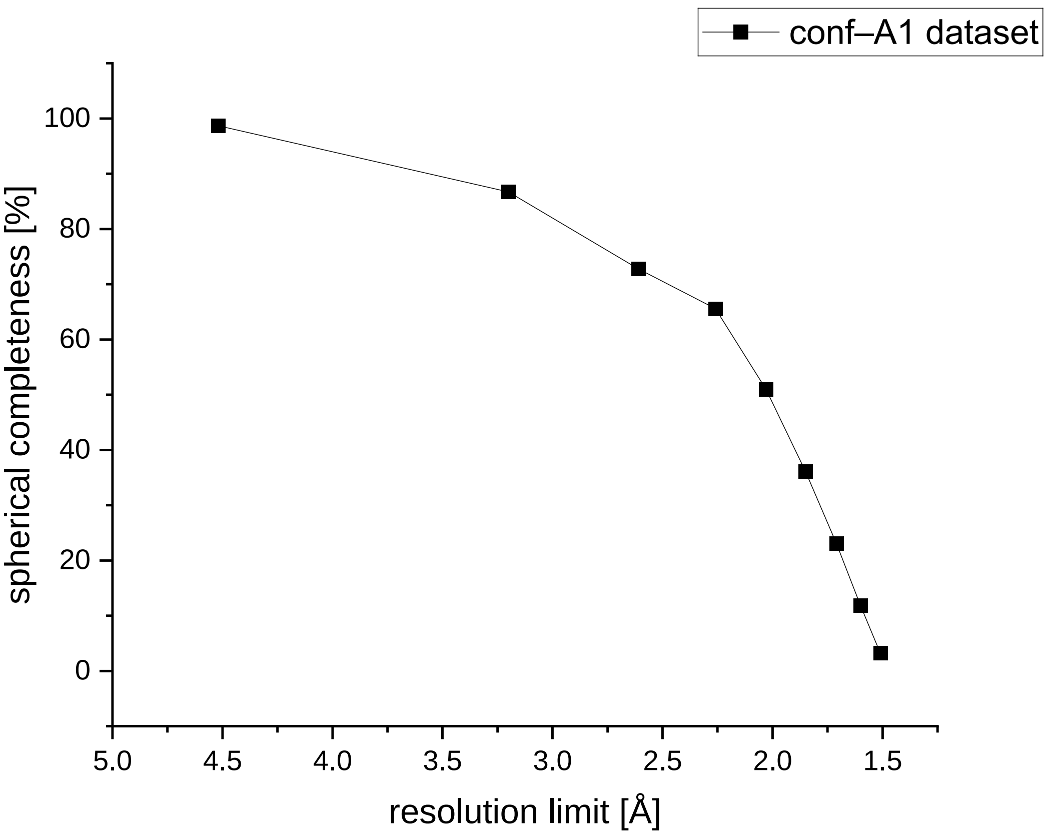

Supplementary Figure 2. Decrease in completeness with increasing resolution. Completeness (spherical) of conf-A1 diffraction data at various resolution limits.

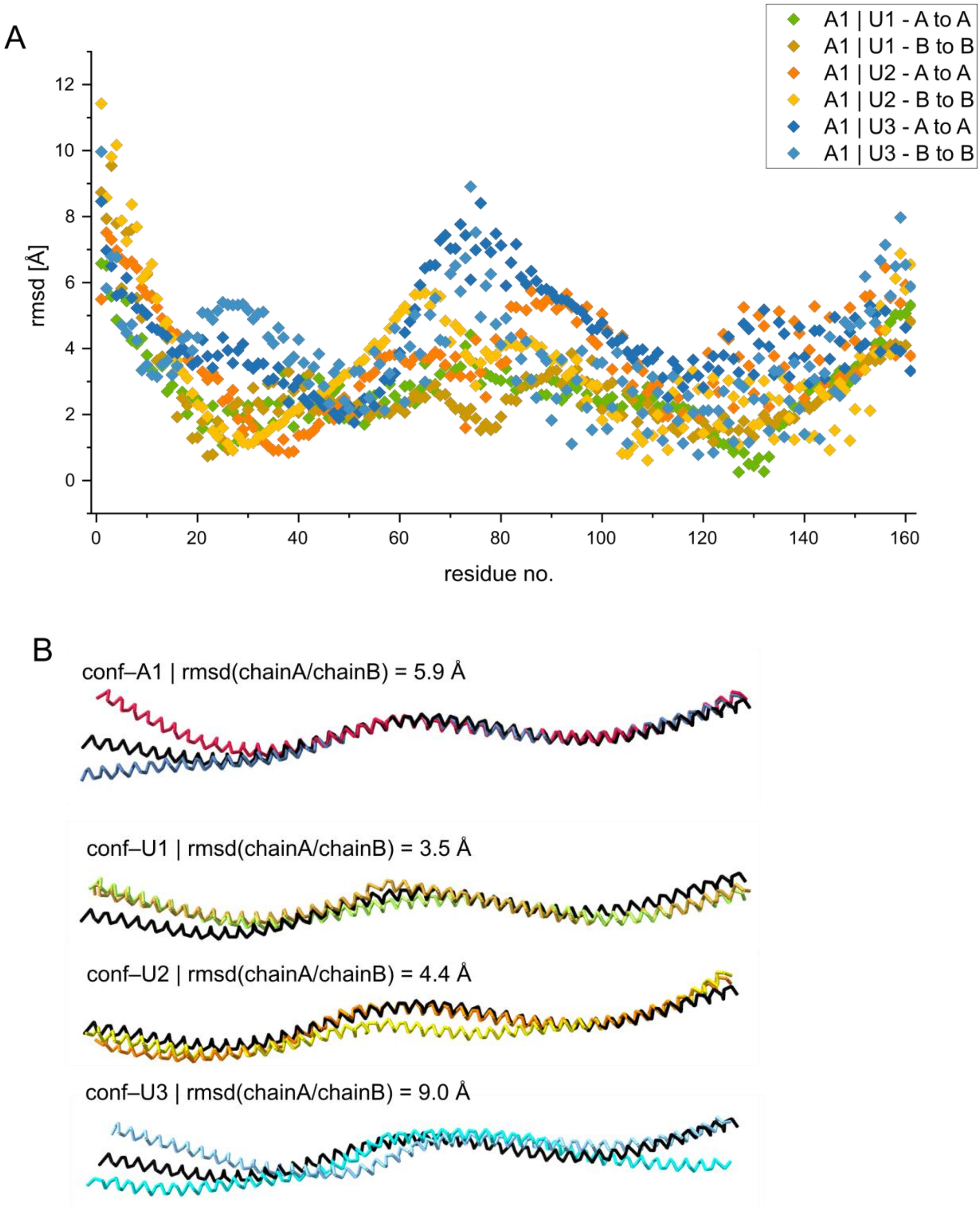

Supplementary Figure 3. RMSD analysis and structural comparison of coiled-coil conformations. *A*, RMSD per residue between C-alpha atoms of conf-A1 and conf-U1, conf-U2, and conf-U3 for each chain individually. *B*, The model of an ideal coiled-coil generated using CCBuilder (black) is superimposed with chains A and B from conf-A1 (blue, red), conf-U1 (gold, green), conf-U2 (yellow, orange) and conf-U3 (blue, light blue).

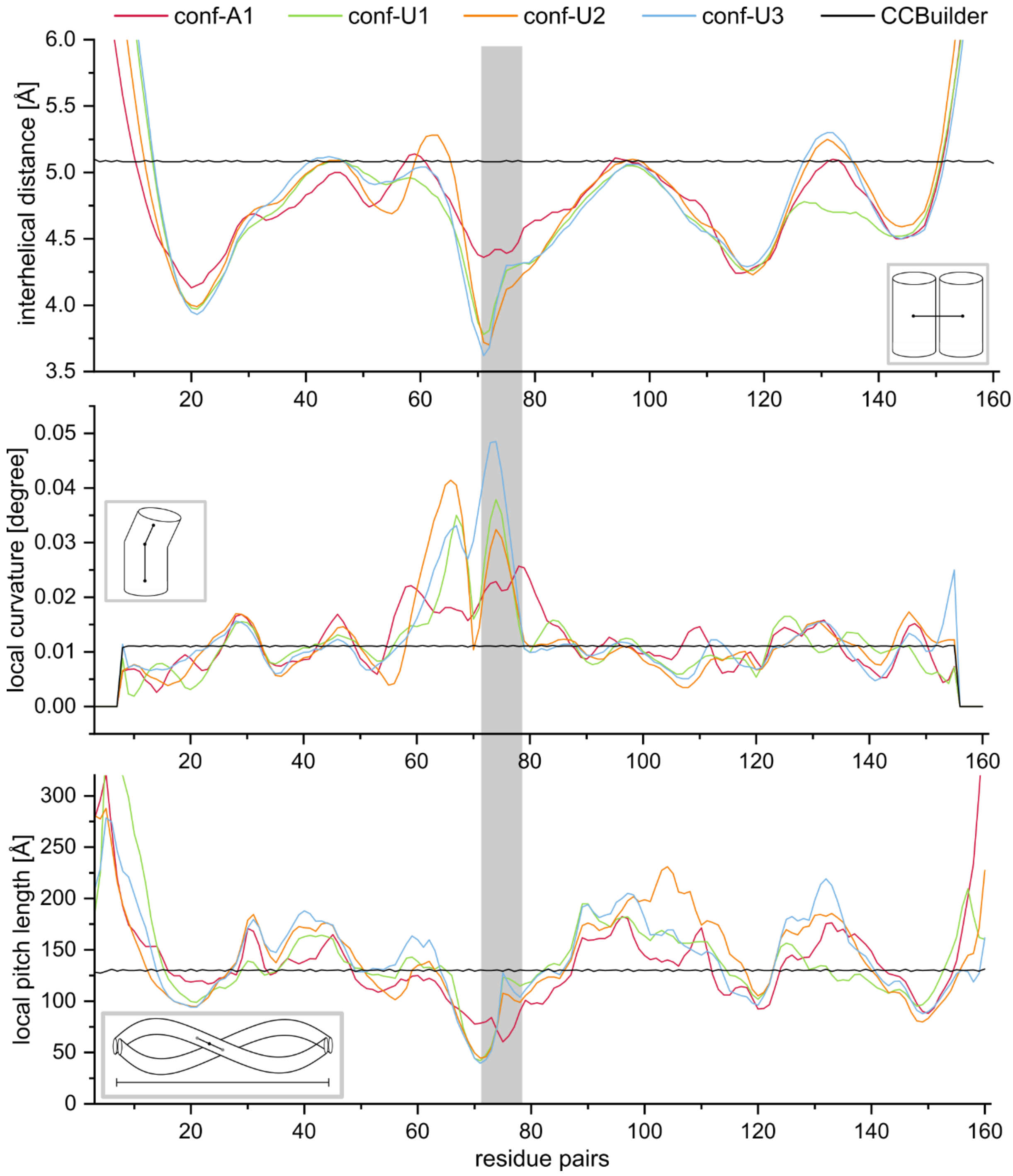

Supplementary Figure 4. Comparative analysis of interhelical distance, local curvature, and pitch length. Interhelical distance, local curvature and local pitch length of conf-A1, conf-U1, conf-U2 and conf-U3 are shown per residue. Parameters for the Tpm^{Cdc8} coiled-coil model generated by CCBuilder are shown in black.

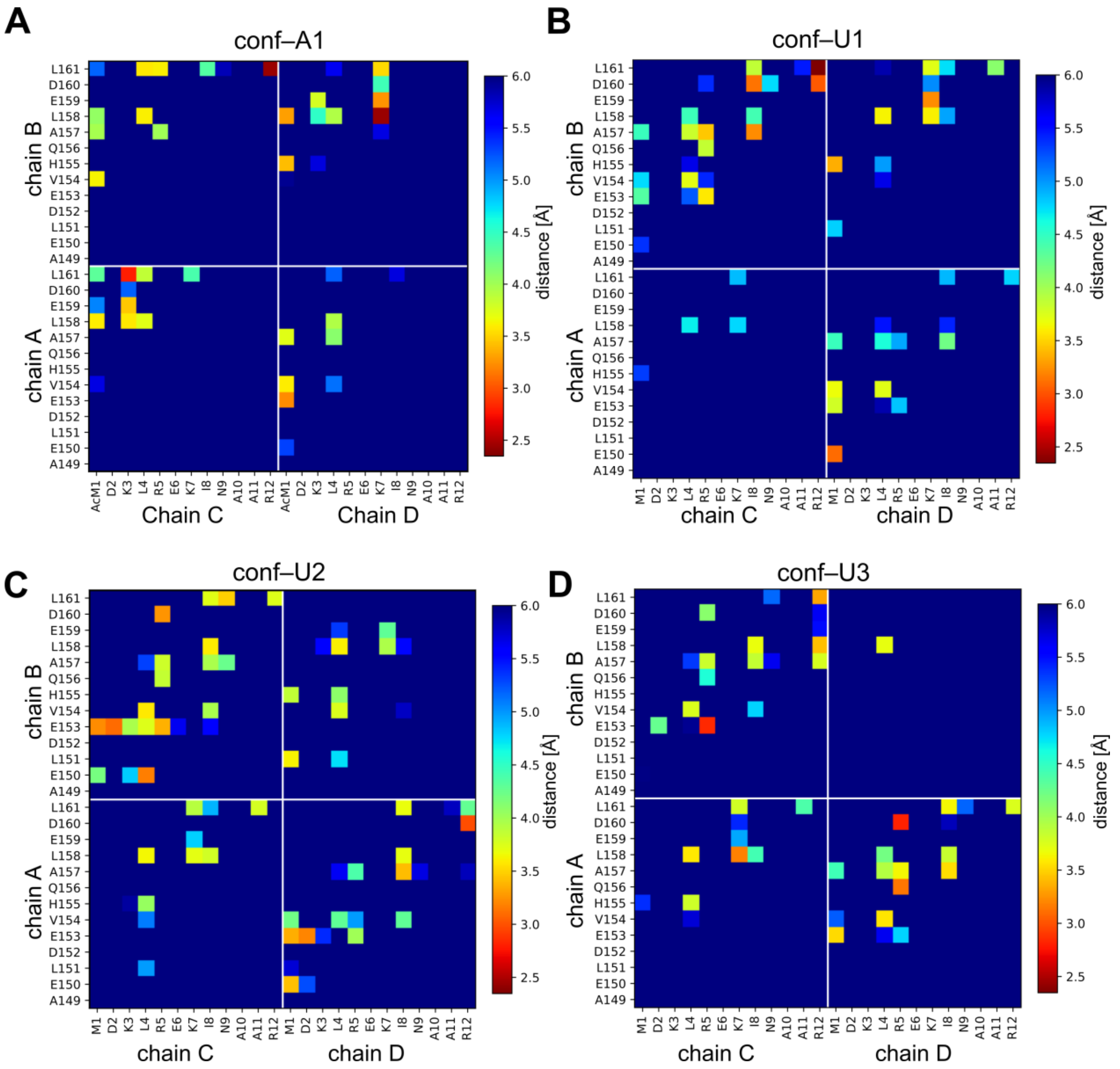

Supplementary Figure 5. **Contact maps for residues within the overlap junction.** Distance maps depicting the minimal distances (with a cutoff of 6 Å) between N-terminal and C-terminal residues in the overlap junction for: *A,* conf–U1, *B,* conf–A1, *C*, conf–U2, and *D*, conf–U3.

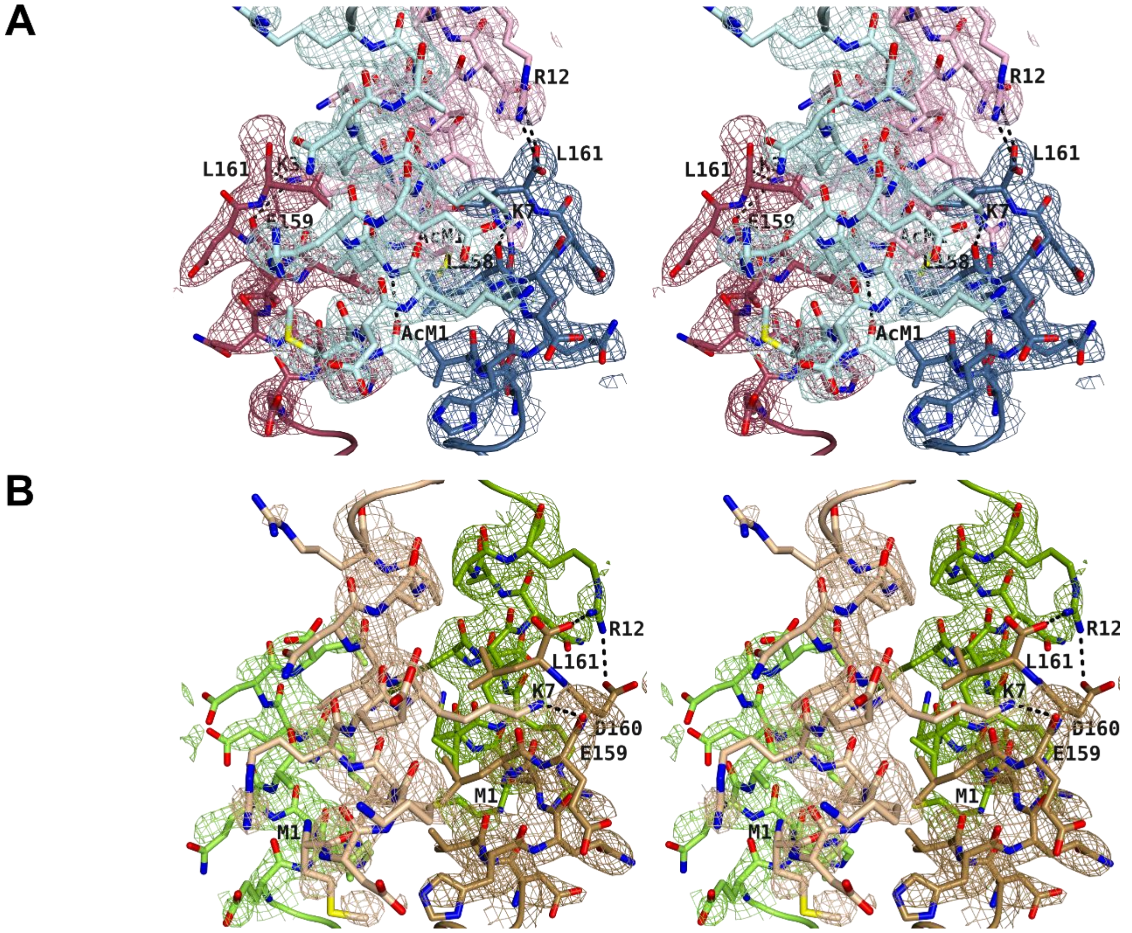

Supplementary Figure 6. **Structural details and interactions in the overlap junctions formed by conf-A1 and conf-U1.** *A*, Stereo view of the overlap junction for conf-A1. *B*, Stereo view of the overlap junction for conf-U1. 2F_o-F_c electron density is shown at a 1.0 σ level for all four protein chains. Salt bridges and hydrogen bonds are shown as dashed lines, with the involved residues labeled. Acetylated methionine 1 in conf-A1 is labeled as AcM1.

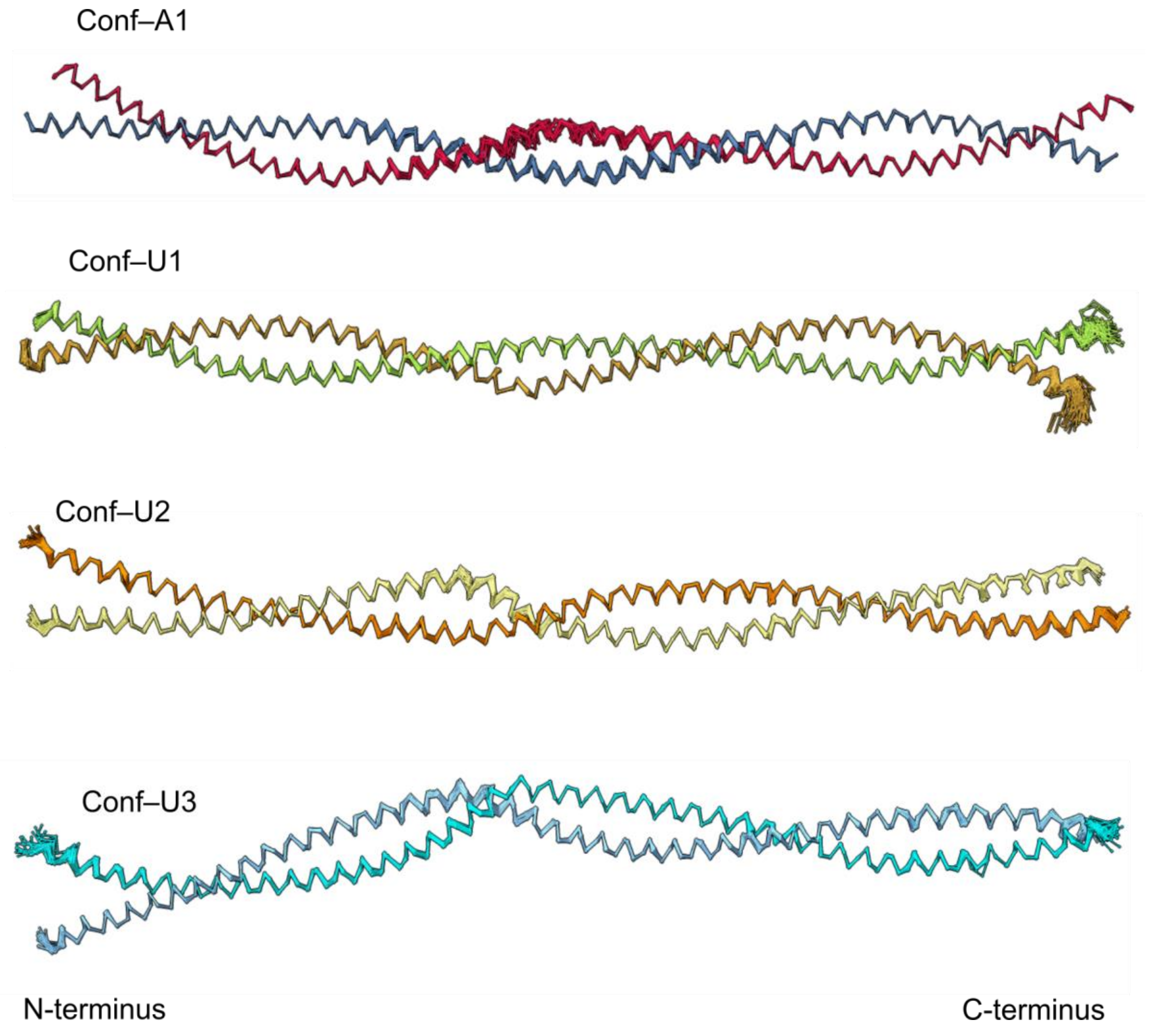

Supplementary Figure 7. **Overlay of ensemble-refined structures.**

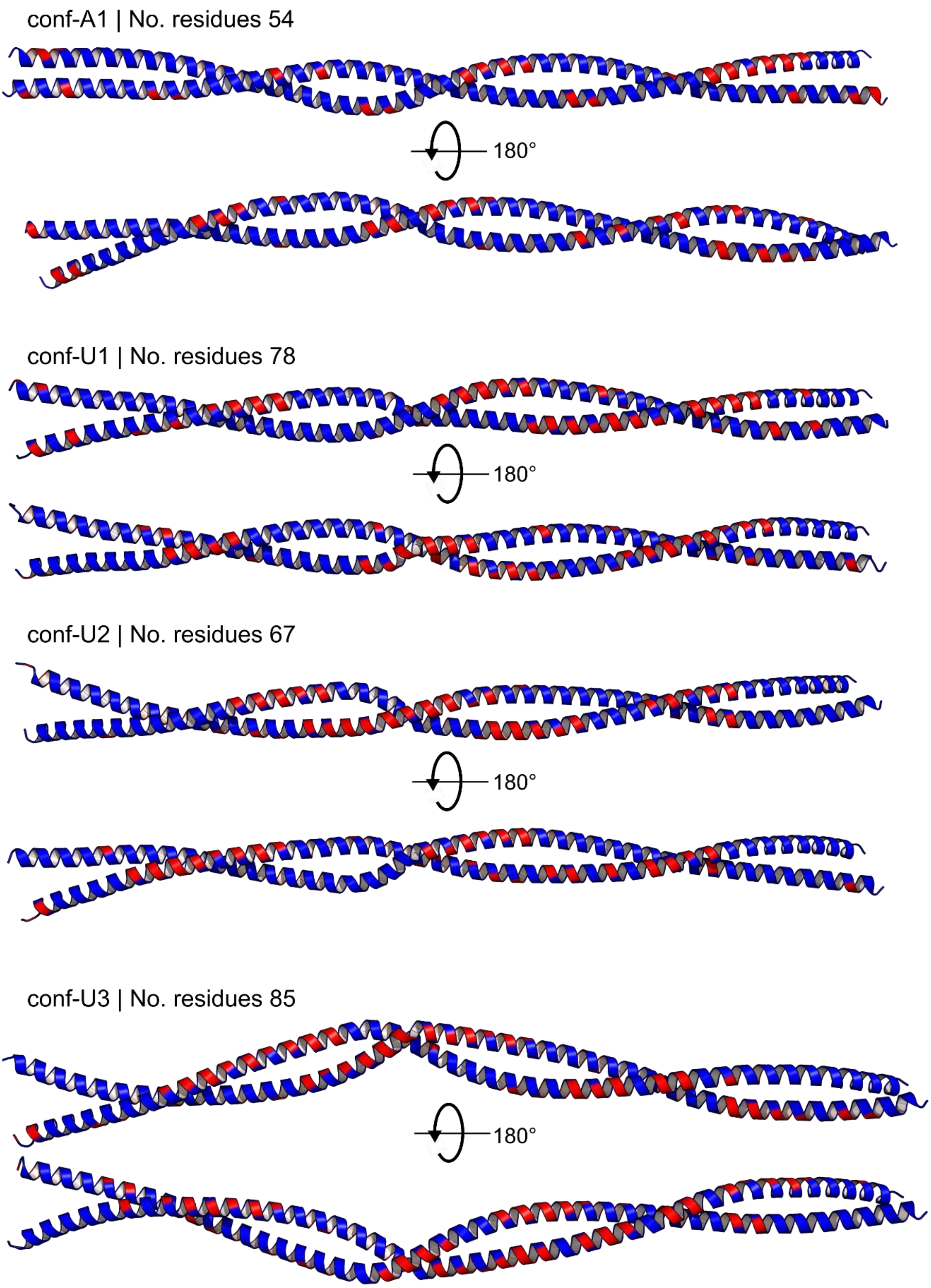

Supplementary Figure 8. **Crystal contacts from Tpm^{Cdc8} stacking in the unit cell.** Positions where crystal contacts occur are colored in red. Each conformer is shown from both sides, with the N-terminus on the left. The number of amino acids involved in crystal contacts is indicated.
